# Congenital giant left atrial appendage aneurysm: a case report

**DOI:** 10.1186/s13019-017-0576-6

**Published:** 2017-03-16

**Authors:** Yan Chen, Yun Mou, Li-Jun Jiang, Shen-Jiang Hu

**Affiliations:** 1 Echocardiography and Vascular Ultrasound Center, The First Affiliated Hospital, College of Medicine, Zhejiang University, Hangzhou, China; 2 Department of Thoracic Cardiovascular Surgery, The First Affiliated Hospital, College of Medicine, Zhejiang University, Hangzhou, China; 3 Institute of Cardiology, The First Affiliated Hospital, College of Medicine, Zhejiang University, No.79, Qing-Chun Road, Hangzhou, China

**Keywords:** Left atrial appendage aneurysm, Echocardiography, Pregnancy, Case report

## Abstract

**Background:**

Congenital left atrial appendage aneurysm (LAAA) is a rare cardiac anomaly with potentially serious complications, including life-threatening systemic thromboembolism, atrial tachyarrhythmia, and cardiac dysfunction. Currently, early surgical intervention is generally recommended to prevent these complications.

**Case presentation:**

We present a case of congenital giant LAAA in a female patient who successfully completed pregnancy and underwent caesarean section with no obvious complications. Surgical resection of the LAAA was performed 3 years later, at the onset of chest pain resulting from compression of adjacent cardiac structures by the LAAA.

**Conclusion:**

Surgical resection is recommended for the majority of patients with LAAA because of potential LAAA-related severe outcomes. However, clinical monitoring may be an optional strategy for asymptomatic patients without intra-atrial thrombus or other complications. Precise evaluation with echocardiography and brain magnetic resonance imaging is valuable for the subsequent management of LAAA.

## Background

Congenital left atrial appendage aneurysm (LAAA) is a rare cardiac malformation that has been reported occasionally since its first description in 1960 [1]. Despite the congenital cause, clinical symptoms usually do not arise until the second to third decades of life. Some LAAA cases are detected incidentally, but most are diagnosed when patients present with palpitation, dyspnea, angina, or thromboembolic events [2–4]. Because LAAA predisposes to thromboembolism, arrhythmias, and heart failure, surgical resection is generally recommended [5]. We describe a case of a 36 year old woman who underwent surgical resection of an LAAA after being diagnosed 3 years prior at the time of pregnancy.

## Case presentation

A 36-year-old female patient with a history of “cardiac cyst (70 × 30 mm)” presented to our hospital for treatment of recurrent episodes of chest pain. The “cardiac cyst” was discovered 3 years earlier by echocardiography when the patient experienced slight dyspnea and palpitation at 37 weeks’ gestation. At that time, the electrocardiogram was normal, and blood coagulation function tests revealed slightly increased fibrinogen and D-dimer levels (4.10 g/L and 1252 μg/L, respectively). Before parturition, these values were 4.07 g/L and 1875 μg/L, respectively. Because uterine inertia could not be resolved during natural delivery, cesarean section was performed. Delivery was successful, and the patient was asymptomatic. Annual check-ups during the following 3 years revealed no abnormity other than an enlarged heart shadow on chest radiographs. Anticoagulation therapy was not administered during the 3-year follow-up period.

**Fig. 1:**
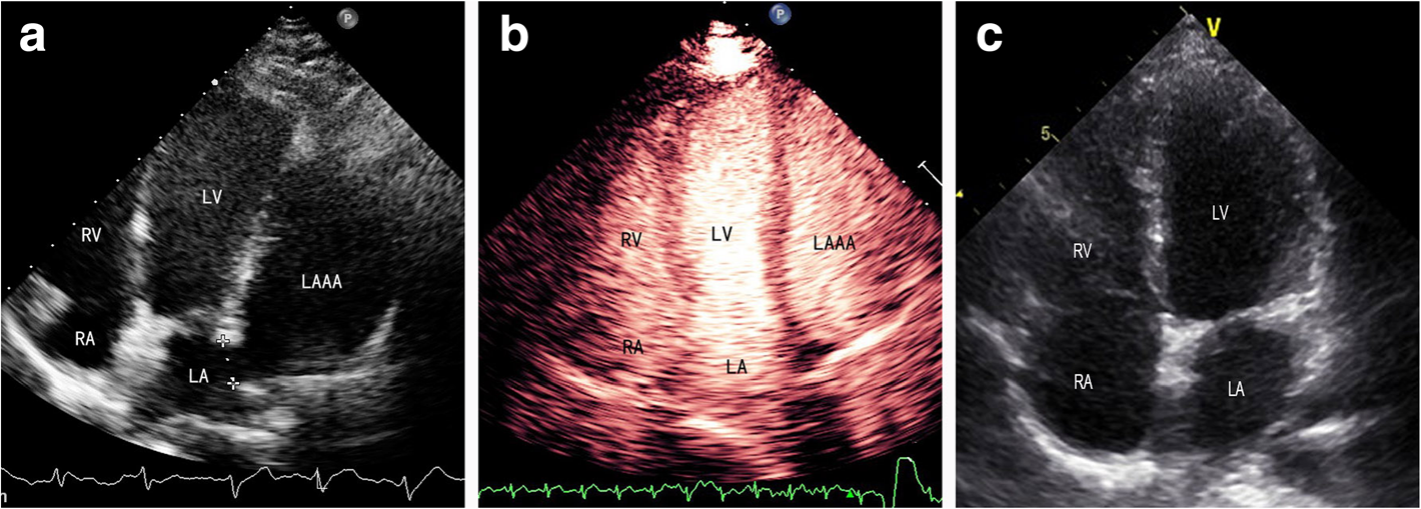
Transthoracic echocardiography showing the appendage aneurysm. **a** Conventional echocardiography showing a giant LAAA compressing the LV. **b** No thrombus was detected in the aneurysm by contrast echocardiography. **c** Echocardiography showing a normal-sized LA and that the aneurysm was no longer present

**Fig. 2:**
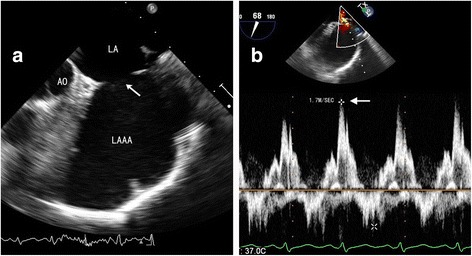
Details of the aneurysm on transesophageal echocardiography. **a** A clear orifice (*arrow*) is present between the aneurysm and the LA. **b** Pulsed wave Doppler imaging showing blood flow at the orifice. Flow velocity was approximately 1.7 m/s

**Fig. 3:**
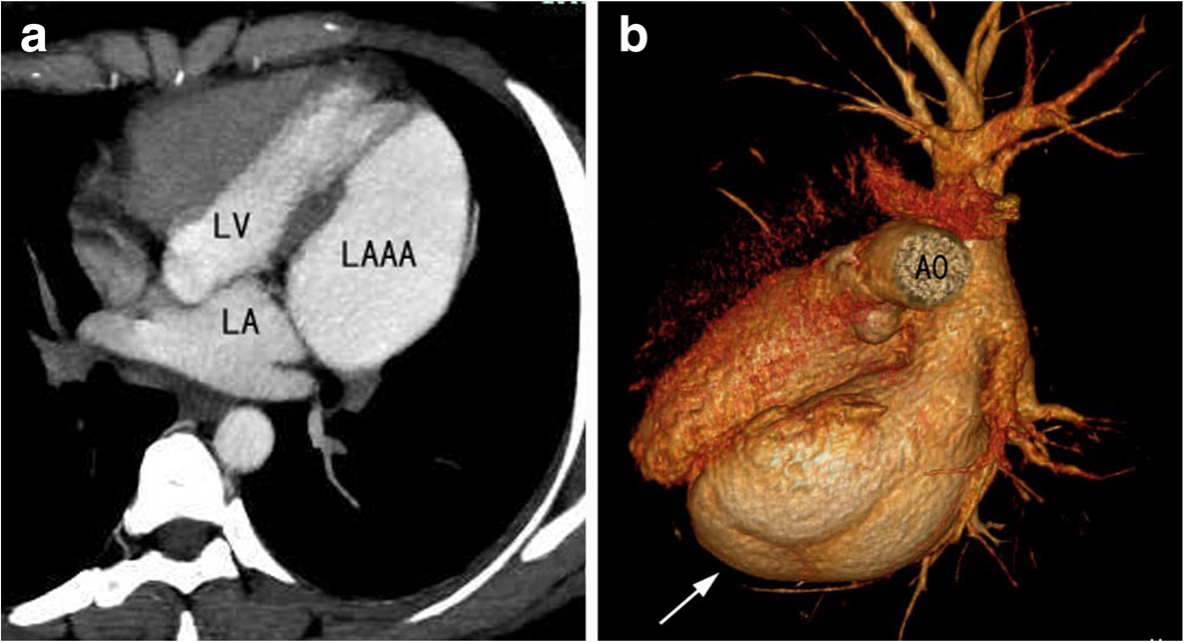
LAAA on CT images. **a** Thoracic CT showing the relationship between the appendage aneurysm and the left heart. **b** Three-dimensional CT image showing the spatial relationship between the LAAA (*arrow*) and the pulmonary veins and left heart. The LAAA was much larger than the LV

Two months before presentation at our hospital, the patient developed gradually worsening chest pain. The electrocardiogram remained normal; however, transthoracic echocardiography (iE Elite, Philips Healthcare, Bothell, WA, USA) revealed a long oval structure attached to the left atrium (LA) adjacent to the left ventricle (LV) and compressing the anterolateral left ventricular wall during the entire cardiac cycle (Fig. 1a). No other structural abnormalities were observed. Contrast echocardiography showed that the long oval structure and the LA were enhanced simultaneously with no filling defect (Fig. 1b). Transesophageal study confirmed a 3.9-cm^2^ channel between the LA and the structure, which measured 96 mm in length and 55 mm wide (Fig. 2a). Migration of blood along the channel was observed with Doppler imaging (Fig. 2b). LAAA was diagnosed definitively, with no thrombi or spontaneous echo contrast observed. Computed tomography (CT) was performed to evaluate the status of the pulmonary veins and other surrounding structures (Fig. 3a, 3b).

**Fig. 4:**
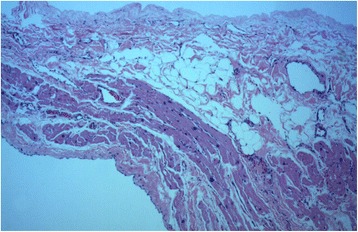
Histology showing the wall of the aneurysm composed of myocardium and fibrotic tissue (hematoxylin and eosin, ×50)

The patient was hospitalized and scheduled to undergo cardiac surgery. Preoperative blood testing, including coagulation function tests, were normal. Median sternotomy was performed, exposing the giant LAAA with intact overlying pericardium. During cardiopulmonary bypass, the base of the LAAA was simply clamped and then resected with a stapler (Echelon 60, Johnson & Johnson, Guaynabo, Puerto Rico, USA). No thrombus within the LAAA or other cardiac anomaly was detected. Anatomical pathology identified the LAAA as a thin-walled and dilated aneurysm of the left atrial appendage. Histopathology described the wall as being composed of myocardium and fibrotic tissue, indicating a true atrial aneurysm (Fig. 4). The patient’s postoperative course was uneventful. At the 6-month follow-up, the patient remained asymptomatic and in sinus rhythm, and transthoracic echocardiography (Vivid E9; GE Healthcare, Strandpromenaden, Horten, Norway) revealed good correction of the LAAA and a normal size for the LA, with no evidence of abnormality (Fig. 1c).

## Discussion

Congenital aneurysm of the atrium is rare and occurs in either the left or right atrium, including bilaterally [5, 6]. To date, approximately 50 congenital LAAA cases have been reported in the literature. The condition usually occurs in the absence of other cardiac defects, except in very rare cases, for example, that associated with tricuspid atresia [7]. LAAA called as ‘giant’ is generally longer than 5 cm. The precise rate of growth of LAAA has not yet been documented; however, size likely increases with patient age. Growth may result from poor myocardial contractility of dysplastic pectinate muscles [8], which changes the LAA from a pump to a reservoir and leads to progressive dilation of the weakening LAA resulting from elevated internal pressure. Obvious enlargement was seen in our case over the 3-year follow-up period. The quickly-enlarging aneurysm can not only compress the adjacent cardiac structures, but can also cause clinical symptoms including palpitation, dyspnea, arrhythmia, and thromboembolic events, frequently arising in the third decade of life [5]. Neonates and infants are more prone to congestive heart failure and respiratory distress with large aneurysms because the condition may be associated with physical compression of the pulmonary veins and airway obstruction, respectively [9, 10]. Although LAAA has been reported in all age groups, to the best of our knowledge, ours is the first report of congenital giant LAAA in a pregnant woman without obvious complications.

LAAA carries a risk of life-threatening complications, including atrial tachyarrhythmia, systemic embolism, myocardial dysfunction and heart failure, and early surgical intervention is generally recommended, even in asymptomatic cases [11]. However, a conservative strategy has been reported in some cases with giant LAAA. Plonska-Gosciniak et al. reported a case of a 45-year-old patient with a giant LAAA (112 × 49 × 80 mm) who presented with exercise intolerance and palpitation and who refused surgical resection but accepted drug management. The patient remained stable with occasional supraventricular arrhythmia at the 20-year follow-up [2]. Sharma et al. reported a case of a 35-year-old patient with LAAA (51 × 66 mm) who declined surgery; the patient’s shortness of breath had resolved at the 6-month follow-up with oral anticoagulation treatment, alone [3]. These findings indicate that early surgical treatment may not be essential to prevent complications provided there are no thromboembolic events and no thrombi are present in the LAAA, as with our patient during pregnancy. Systemic embolism is a common complication of untreated LAAA [11]. Although no symptomatic thromboembolic events occurred in our case, we could not determine whether there were silent events without performing brain magnetic resonance imaging (MRI). The LAAA in our case was so large, it was an obvious potential source of embolism; therefore, performing brain MRI in the follow-up period is necessary, especially for asymptomatic cases.

Drug management may be an option for LAAA patients with mild cardiac symptoms, but not for those with symptoms caused by thrombosis or structural compression. Although this conservative strategy may increase the risk of complications, cardiac surgery also has inherent challenges. Using drug management, monitoring changes in the LAAA and LA are necessary because dilation of the LAAA may trigger atrial tachyarrhythmia [12] and can compress adjacent cardiac structures such as the coronary artery or ventricular wall, as in our case. However, no study has defined the size of LAAA that induce these complications. A recent study demonstrated that aneurysm size did not predict thrombus formation or embolic events, but that atrial tachyarrhythmia did [5]. Therefore, observation of clinical symptoms is also essential.

Although conservative approaches relieved symptoms in a small number of cases, surgery is required for neonates and infants, as well as adults, with severe complications or other co-existing abnormalities. Aneurysmectomy is usually performed via median sternotomy, which is more suitable than other approaches for removal of a large LAAA with thrombus, and in patients undergoing concurrent mitral correction, in whom regurgitation occurs mainly as a consequence of distortion of the mitral annulus by the aneurysm [10]. Although endoscopic resection may be appropriate for smaller congenital aneurysms, the technique increases surgical difficulty and may be more popular in otherwise healthy young patients. The Cox–Maze III procedure is considered prudent in cases of atrial enlargement, basal aneurysmal dilatation, or persistent atrial fibrillation [13]. After surgery, there are almost no reports of recurrent symptoms, except in one patient who remained in atrial fibrillation after numerous attempts at defibrillation [4]. In our case, cardiac surgery was the only choice to relieve the compression of the ventricular wall. The Maze procedure was not performed in our case because there was no atrial enlargement or atrial arrhythmia.

Precise evaluation of LAAA can provide important information for subsequent management. Transthoracic echocardiography is considered an adequate and primary method to identify LAAA, thrombi, and other cardiac abnormalities. However, transesophageal echocardiography, which provides more detail and clear visualization, including blood flow across the orifice and tiny thrombi, is concurrently recommended for precise delineation of the appendage. When the extent of the giant LAAA cannot be visualized well on echocardiography, CT and MRI can provide more accurate anatomic definition, especially in ruling out differential diagnoses. We performed contrast echocardiography to exclude the possible existence of thrombi. This method may be more suitable for a giant LAAA with an irregular form [13]. Also, progressively declining flow velocities through the orifice is a dangerous signal of blood stasis in LAAA [14]. In these cases, spontaneous echo contrast should be carefully evaluated because of the increased risk of intra-atrial thrombus formation secondary to stasis.

LAAA can combine with other cardiac abnormalities, and because of differences in appropriate management, it is vital to precisely evaluate the entire cardiac structure with echocardiography. Proposed diagnostic criteria for congenital LAAA are: (1) origin from an otherwise normal LA, (2) clearly-defined communication with the LA, (3) location within the pericardium, and (4) distortion of the free wall of the LV by the aneurysm [15]. Although LAAA is very rare, it can be life-threatening if thromboembolism induces cerebrovascular incidents. When young patients present with the above-mentioned symptoms without other related disease, the possibility of LAAA should be considered.

## Conclusion

LAAA is a rare defect that is associated with cardiovascular morbidity and mortality by predisposing to cardiac dysfunction, atrial tachyarrhythmia, and thromboembolism, and surgical resection is generally recommended to prevent devastating complications. Conservative approaches may be an optional strategy for certain patients without intra-atrial thrombi in the symptom-free period, especially with a small LAAA detected incidentally. Comprehensive evaluation of echocardiography, brain MRI, and clinical symptoms may be helpful for the subsequent management of LAAA.

## Abbreviations

AO: Aorta
CT: Computed tomography
LA: Left atrium
LAAA: Left atrial appendage aneurysm
LV: Left ventricle
MRI: Magnetic resonance imaging
RA: Right atrium
RV: Right ventricle
